# Proceedings of the 2018 MidSouth Computational Biology and Bioinformatics Society (MCBIOS) conference

**DOI:** 10.1186/s12859-019-2618-7

**Published:** 2019-03-14

**Authors:** Jonathan D. Wren, Robert J. Doerkson, Inimary T. Toby, Bindu Nanduri, Ramin Homayouni, Prashanti Manda, Shraddha Thakkar

**Affiliations:** 1 Arthritis and Clinical Immunology Research Program, Division of Genomics and Data Sciences, Oklahoma Medical Research Foundation, 825 N.E. 13th Street, Oklahoma City, OK 73104-5005 USA; 2 Biochemistry and Molecular Biology Department, University of Oklahoma Health Sciences Center, Oklahoma City, USA; 3 Stephenson Cancer Center, University of Oklahoma Health Sciences Center, Oklahoma City, USA; 4 Department of Geriatric Medicine, University of Oklahoma Health Sciences Center, Oklahoma City, USA; 5 Department of BioMolecular Sciences, Division of Medicinal Chemistry, School of Pharmacy, The University of Mississippi, University, Jackson, MS 38677 USA; 6 Department of Biology, University of Dallas, 1845 E Northgate Dr, Irving, TX 75062-4736 USA; 7 Department of Basic Sciences, College of Veterinary Medicine, Mississippi State University, Mississippi State, MS 39762 USA; 8 Foundational Medical Sciences, University of Oakland William Beaumont School of Medicine, Rochester, MI 48309-4482 USA; 9 Department of Computer Science, University of North Carolina at Greensboro, Greensboro, USA; 10 Division of Bioinformatics and Biostatistics, National Center for Toxicological Research, US Food and Drug Administration, Jefferson, AR 72079 USA

**Keywords:** Bioinformatics, Conferences, MCBIOS, ISCB

## Introduction

The XVth Annual MidSouth Computational Biology and Bioinformatics Society (MCBIOS XV) conference was held in Starkville, MS from March 29–31, 2018 at Mississippi State University (MSU) within the Mill Conference Center. MSU had previously hosted the conference (MCBIOS VI) in 2009. The theme of MCBIOS XV was “Genomics and Big Data”. The co-chairs and conference hosts were Drs. Bindu Nanduri, Andy Perkins, and Daniel G. Peterson from MSU. The program was co-chaired by Dr. Shraddha Thakkar from the National Center for Toxicological Research (NCTR) within the US Food and Drug Administration (FDA), Dr. Mary Yang, from University of Arkansas at Little Rock (UALR) and Dr. Prashanti Manda from University of North Carolina at Greensboro.

The conference was attended by 183 registered participants, of these, 73 registered participants were in the professional category, while 13 were postdoctoral fellows and 97 were student participants. A total of 157 abstracts were submitted for MCBIOS XV, including 65 oral presentations and 92 poster presentations at the meeting. There were nine breakout sessions conducted during the meeting. Each breakout session included a presentation by a featured speaker, a renowned scientist in the topic of that session, followed by four additional presentations in that area. Dr. Cesar M. Compadre, from University of Arkansas for Medical Sciences served as the finance coordinator for the conference. Dr. Ping Gong, at the US Army Engineer Research and Development Center, Vicksburg served as the coordinator of Young Scientist Research Excellence Award. Dr. George Popescu from the Institute of Genomics, Biocomputing and Biotechnology (IGBB) of MSU served as the poster session coordinator, and Dr. William S. Sanders from The Jackson Laboratory served as the workshop coordinator.

For 2019–20, Dr. Weida Tong, Director of Division of Bioinformatics and Biostatics from NCTR/FDA was chosen as the President-Elect and Dr. Ramin Homayouni from University of Memphis as the President.

- **Keynote speakers for MCBIOS 2018**
- **Keynote Session I:** “Next-Gen Data Science”, Russ Wolfinger, Ph.D., Director of Scientific Discovery and Genomics, JMP Life Sciences, SAS Institute, Cary, NC
- **Keynote Session II:** “Real World Data and Precision Medicine: Treatment Selection and Dose Optimization Strategies”, Lawrence J. Lesko Ph.D., F.C.P., University of Florida, Orlando, FL
- **Keynote Session III:** “No-Boundary Thinking: Defining Problems So Their Solutions Matter”, Steve Jennings, Ph.D., UALR
- **Keynote Session IV**: **“**Informatics Tools for Big Biologicals and Small Drug Molecules”, William J Welsh, Ph.D., Norman H. Edelman Professor in Bioinformatics, Rutgers University, New Brunswick, NJ
- **Keynote Session V:** “A decade of MAQC effort and its contribution to our understanding of high-throughput genomics technologies”, Weida Tong, Ph.D., Director, Division of Bioinformatics and Biostatistics, NCTR/FDA

- **The conference program included three workshops:**
- Workshop I: “Advanced Data Analytics using JMP Genomics”, Wenjun Bao, Ph.D., JMP Life Sciences, SAS Institute, Cary, NC.
- Workshop II: “Career Development Workshop for Young Scientist”, Inimary Toby, Ph.D., University of Dallas, Dallas, TX.
- Workshop III: “MCBIOS and No-Boundary Thinking Joint Bioinformatics Research workshop”.
  Session chair - Steve Jennings, Ph.D., UALR.
- Presentations:
  “Encoding biomedical knowledge using hetnets”, Daniel Himmelstein, Ph.D., University of Pennsylvania, Philadelphia, PN.
  “Microbial interactions and microbe-host interactions”, Hongmei Jiang Ph.D., Northwestern University, Evanston, IL.
  “Evolution as a metaphor for No Boundary Thinking”, Scott M. Williams, Ph.D., Case Western Reserve University, Cleveland, Ohio.
- Panel Discussion:
- Joan Peckham, Ph.D., University of Rhode Island
- Xiuzhen Huang, Ph.D., Arkansas State University
- Scott M. Williams, Ph.D., Case Western Reserve University
- Hongmei Jiang, Ph.D., Northwestern University
- Daniel Himmelstein, Ph.D., University of Pennsylvania
  In addition to the workshops, MCBIOS provided assistance to students in preparing their resume. A one-on-one resume clinic was conducted by Gladys Awosemo, HRP, Baylor Scott and White Health, TX.
  **Breakout Session I:** Plant Omics I.
  Session Chair – Sorina C. Popescu, Ph.D., Assistant Professor, MSU.
  Featured speaker: Marilyn Warburton, Ph.D., United States Department of Agriculture, Agriculture Research Services, MS, “A pathway-based method to interpret GWAS results”.
  **Breakout Session II:** Next generation tools for environment and health research.
  Session Chair and featured speaker: Natalia Reyero, Ph.D., US Army Engineer Research and Development Center, Vicksburg MS, “Next Generation Tools for Environmental Research”.
  **Breakout Session III:** Drug Discovery and Precision Medicine.
  Session Chair and featured speaker: Robert J. Doerksen, Ph.D., University of Mississippi (UM), Oxford, MS, “Protein structure-based virtual screening: deep learning for precision medicine”.
  **Breakout Session IV:** Breakout Session IV: Plant Omics II.
  Session Chair – Sorina C. Popescu, Ph.D., MSU.
  Featured Speaker: Tessa Burch-Smith, Ph.D., University of Tennessee, Knoxville, TN,
  “Focused ion beam-scanning electron microscopy for three-dimensional modelling of cellular ultrastructure”.
  **Breakout Session V:** Transcriptomics and Genome Sequencing.
  Session Chair and featured speaker: Brian Counterman, Ph.D., MSU, “Patternize: an R package for color pattern variation”.
  **Breakout Session VI:** Big Data and Risk Assessment.
  Session Chair – Minjun Chen, Ph.D., NCTR/FDA.
  Featured Speaker: William Mattes, Ph.D., NCTR/FDA, “Systems Biology and Big Data: Little Mitochondria as a Big Example”.
  **Breakout Session VII:** Genomics and Proteomics application.
  Session Chair - Zhichao Liu, Ph.D., NCTR/FDA.
  Featured Speaker: Rakesh Kaundal, Ph.D., Utah State University, Logan, UT, “Complete genome sequence of *Pythium brassicum* P1, an oomycete root pathogen: insights into its host specificity to Brassicaceae”.
  **Breakout Session VIII:** Genomics and Infectious Disease.
  Session Chair and Featured speaker: Stephen Pruett, Ph.D., MSU, “Machine Learning Analysis of the Relationship between Changes in Immunological Parameters and Changes in Resistance to *Listeria monocytogenes*: A New Approach for Risk Assessment and Systems Immunology”.
  **Breakout Session IX:** MCBIOS Group Projects.
  Shraddha Thakkar, Ph.D., NCTR/FDA.
  William Sanders, Ph.D., IT Research Cyberinfrastructure, The Jackson Laboratory, Bar Harbor, ME.

**Best Paper Award, MCBIOS 2018**: Phillip Berg et al., “Evaluation of Linear Models and Missing Value Imputation for the Analysis of Peptide-Centric Proteomics” [1].

**Best Paper Runner-up, MCBIOS 2018**: Bohu Pan et al., “Similarities and differences between variants called with human reference genome HG19 or HG38” [2].

This was the 2nd year for “MCBIOS Young Scientist Excellence Award” awards to recognize students and postdoctoral fellows that exhibit scientific excellence in the field of Bioinformatics. Student and postdoctoral fellows went through a rigorous award application process with both internal and external judges. The top five candidates were selected to present during the opening session on March 29th.

To compete, applicants submitted an extended abstract with a description of the innovation of their research and their specific contribution to the work presented, from which the quality and impact of the research was judged. Initiative in expanding their skills and bringing multidisciplinary talent to their project was an important consideration for selection for an oral presentation, and the quality of the presentation during the plenary session was the primary consideration for award. Additional evaluation criteria included creativity, dedication and multidisciplinary contribution. This award was supported by the FDA grant to MCBIOS (5R13FD005931–03) and JMP Life Sciences.

### MCBIOS young scientist excellence award

#### 2018 Post-doctoral winners

First Place: Sundar Thangapandian, Ph.D., University of Illinois Urbana-Champaign, Urbana, IL.

“Quantitative Target-specific Toxicity Prediction Model (QTTPM): A Novel Computational Toxicology Approach Integrating Molecular Dynamics Simulation and Machine Learning”.

Second Place: Brian Walker, Ph.D., UALR.

“Synthesis of xanthine derivatives for the inhibition of PARG”.

Third Place: Darshan Mehta, Ph.D., NCTR/FDA.

“Mining pharmacogenomic information from drug labeling using FDALabel database for advancing precision medicine”.

#### 2018 Graduate student winners

First Place: Chathurani Ranathunge, MSU.

“Transcribed microsatellites as engines of adaptive evolution in common sunflower”.

Second Place: AyoOluwa Aderibigbe, UM.

“Sifting through big data: the search for peripherally-restricted cb1 receptor antagonists and inverse agonists”.

Third Place: Gouri Mahajan, UM Medical Center, Jackson, MS.

“Altered Neuro-inflammatory Gene Expression in Hippocampus in Major Depressive Disorder”.

#### Poster session award

The poster session was held on March 29th, 2018. Student and post-doctoral posters were judged for presentation quality and scientific content by at least two MCBIOS professional members with no apparent conflict of interest.

#### Poster session student winners

First Prize.

Hunter Porter, the University of Oklahoma Health Science Center and the Oklahoma Medical Research Foundation, Oklahoma City, OK.

“Testing the Limits of the Epigenetic Clock”.

Second Prize.

Bryan Naidenov, Oklahoma State University, Stillwater, OK.

“Exposing the hidden chromatin regulatory framework with recurrent deep learning and genomic sequence data”.

Third Prize.

Ting Li, UALR.

“Voxel-Based Morphometry Study of Alzheimer’s disease”.

#### Poster session post-doctoral winners

Brian Walker, Ph.D., UALR.

“Synthesis of xanthine derivatives for the inhibition of PARG”.

### Selecting papers for the MCBIOS proceedings

From the work presented at MCBIOS 2018 eligible to be considered for inclusion in the Proceedings, a total of 18 papers were submitted. Of these, 12 papers were accepted (67% acceptance rate). All papers were anonymously peer-reviewed by at least two reviewers and those deemed acceptable during peer-review were quantitatively ranked on the basis of three evaluation criteria: Novelty (1–5), Impact (1–5) and Clarity (1–3), to determine awards for Best Paper as well as Runner-Up. Editors that were co-authors of submitted papers were not permitted to handle their own papers editorially. Papers generally fell into three categories:

### Networks and microbial communities

Aleksandra Perz et al. [3] developed a novel software tool for capturing microbial shifts between conditions. Their goal was to improve the ability to identify similar shifts in species abundance across multiple experiments, which is of importance in human metagenome projects. Using data from the American Gut Project (AGP), their algorithm identifies groups of shifts with similar effects on the microbiome and shows that similar dietary interventions display similar microbial abundance shifts.

Thrash et al. [4] developed a standalone publicly available Python program called Keanu, which uses shotgun sequenced metagenomic data to produce interactive taxonomical visualization and quantitation of the organisms in a sample. The visualization can be in the form of a collapsible dendrogram or a bilevel partition graph. The utility of the tool was demonstrated using a soil sample from an archeological site in Alaska.

Sujoy Roy et al. [5] developed a method to evaluate microarray probe quality by estimating the “cohesiveness” of correlated genes within the literature. The premise being that good probes will yield measurements that reflect the biology and that this could be measured by estimating the similarity of documents associated with highly correlated genes. They use SIRT3 as a test case and find supportive evidence.

Zheng Wang et al. [6] implemented and benchmarked a suite of machine learning algorithms for the task of predicting residue-residue contacts. These algorithms include random forests, direct-coupling analysis, support vector machined, and stacked denoising autoencoders. The primary findings indicate that random forests and other ensemble methods combined with support vector machines outperform other algorithms.

### Genomics & Transcriptomics

Bohu Pan et al. [2] reported a systematic comparison of the Single Nucleotide Variant (SNV) identification, using the two most recent versions of the human genome: HG19 and HG38, utilizing multiple aligners and SNV detection algorithms. Based on their in depth comparison of the identified SNVs, they recommend the usage of the newer version (HG38) in SNV analysis.

Visanu Wanchai et al. [7] focus on Barrett’s esophagus (BE) as a predisposing factor for the development of esophageal adenocarcinoma (EAC). Using a case-control framework, they demonstrate that integration of tracts of homozygosity, repetitive elements, and insertion/deletions within exomes can help functionally prioritize factors contributing to low to moderate penetrance predisposition to BE/EAC.

### Imaging/visualization

Bayraktar et al. [8] introduce a novel active contours based skin lesion border detection method for dermoscopy images. This study demonstrates the utility of Smoothed Particle Hydrodynamics kernels and probability maps for increased accuracy of segmentation and removal of the leaking problem, a main concern associated with active contours.

Demriel et al. [9] present a novel approach for Generative Anatomy Modeling Language (GAML) to automatically detect geometric partitions in 3-dimensional anatomy. The primary contribution of this approach is to speed-up prior non-linear optimization GAML models by reducing their exponential time complexity to a linear complexity.

### Miscellaneous

George Popescu et al. [10] worked on the identification of Multispecies MAPKKK analysis and identified that MAPKK reveals conserved MAP3K expansion regions. These MAP3K regions and defines the MAP3K gene family in the *Gossypium hirsutum.*

Phillip Berg et al. [1] developed and evaluated a computational pipeline, written in R, to analyze protein post-translational modifications (PTMs) from proteomics datasets. Using various linear models and imputation of missing values, they show this method is able to outperform simpler tests in the estimation of the fraction of proteins with PTMs.

Hong Fang et al. [11] study subsections of new drugs labelled with the FDA’s disclosure system for Adverse Drug Reactions (ADRs) within specific ADR subsections: The Boxed Warning, Warnings and Precautions, and Adverse Reactions subsections (which vary in their severity) to identify the reported ADRs are in the section most proportional to their severity and, thus, most reflective of cautionary risk.

### Future meetings

The 16th Annual MCBIOS conference will be hosted by the University of Alabama at Birmingham (UAB) from 28th–30th March, 2019. Conference co-chairs are Drs. Jake Chen and Matthew Might from UAB. Program co-chairs are Dr. Weida Tong from NCTR/FDA and Dr. Puri Bangalore from UAB.
